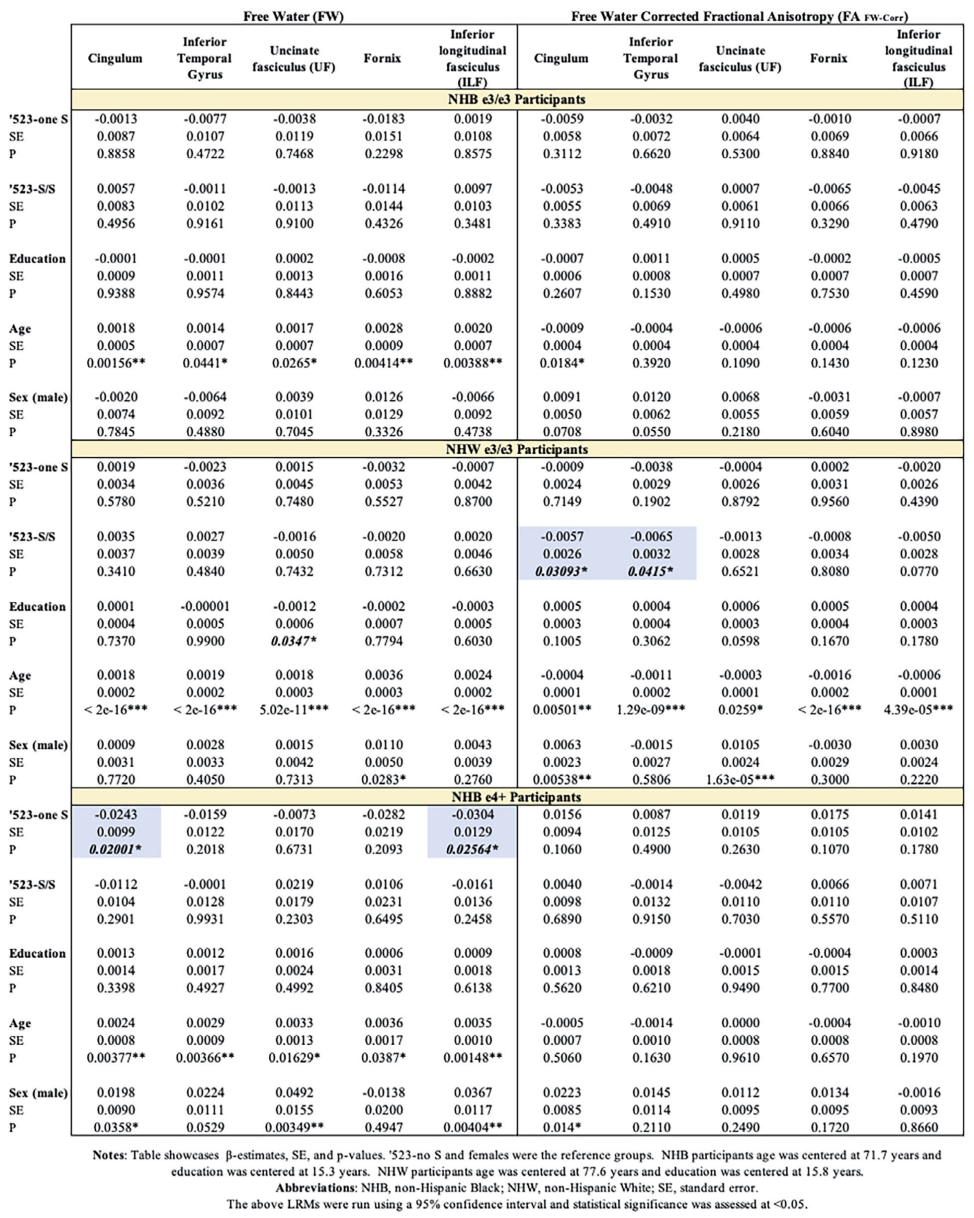# Association of APOE/TOMM40 Haplotypes and Limbic System White Matter Microstructure in Black and White Individuals

**DOI:** 10.1002/alz.091065

**Published:** 2025-01-09

**Authors:** Katelyn Elizabeth Mooney, Derek B. Archer, Timothy J. Hohman, Aditi Sathe, Ose Kadiri, Lisa L. Barnes, Kacie Deters

**Affiliations:** ^1^ University of California, Los Angeles Neuroscience Interdepartmental Program (NSIDP), Los Angeles, CA USA; ^2^ Vanderbilt Memory and Alzheimer’s Center, Vanderbilt University Medical Center, Nashville, TN USA; ^3^ Vanderbilt Genetics Institute, Institute for Medicine and Public Health Vanderbilt University Medical Center, Nashville, TN USA; ^4^ Vanderbilt Memory & Alzheimer’s Center, Department of Neurology, Vanderbilt University Medical Center, Nashville, TN USA; ^5^ Vanderbilt Genetics Institute, Vanderbilt University Medical Center, Nashville, TN USA; ^6^ Vanderbilt Memory & Alzheimer’s Center, Vanderbilt University Medical Center, Nashville, TN USA; ^7^ University of California, Los Angeles, Los Angeles, CA USA; ^8^ Rush University Medical Center, Chicago, IL USA; ^9^ Department of Psychiatry and Behavioral Sciences, Rush University Medical Center, Chicago, IL USA; ^10^ University of California, Los Angeles Integrative Biology and Physiology (IBP), Los Angeles, CA USA

## Abstract

**Background:**

APOE is in linkage disequilibrium with the length of poly‐T repeats at the rs10524523 (‘523) locus of the TOMM40 gene. APOE‐ε3 is associated with short (S) and (VL) variants of ‘523 in white and Black individuals. In white individuals, APOE‐ε4 is associated with the long (L) ‘523 variant, but is associated with ‘523‐S, ‘523‐L, and ‘523‐VL variants in Black individuals. Black ε3‐’523‐S carriers have faster rates of cognitive decline while Black ε4‐’523‐S carriers have slower rates of cognitive decline. Little is known about the association of these haplotypes with white matter integrity. This study aims to determine the association between APOE/TOMM40 haplotypes and white matter microstructure (WMM) in tracts important for cognition and risk of AD.

**Methods:**

Data comes from participants without cognitive impairment from the Rush Memory and Aging Project and the Minority Aging Research Study who self‐identified as non‐Hispanic white (N=500) and non‐Hispanic Black (N=144). WMM was measured using diffusion MRI (dMRI). dMRI was preprocessed using PreQual and microstructural metrics were calculated in DTIFIT and MATLAB. Data was harmonized using Longitudinal ComBat. We assessed free‐water (FW; higher values are abnormal) and FW‐corrected fractional anisotropy (FA_FW‐corr_; lower values are abnormal) in five limbic tracts that are associated with AD: the inferior longitudinal fasciculus, cingulum, fornix, inferior temporal gyrus, and uncinate fasciculus. The effect of 0, 1, or 2 copies of ‘523‐S on FW and FA_FW‐corr_ was assessed using linear regression models, controlling for age, sex/gender, and education.

**Results:**

White ε3/ε3 participants with two copies of ‘523‐S had lower FA_FW‐corr_ in the cingulum (β=−0.006, SE=0.003, P=0.031) and inferior temporal gyrus (β=−0.007, SE=0.003, P=0.042) (Table). Black ε4+ participants with one‐‘523‐S had lower FW in cingulum (β=−0.024, SE=0.01, P=0.020) and inferior longitudinal fasciculus (β=−0.0304, SE=0.013, P=0.026) (Table).

**Conclusions:**

This is the first study examining APOE/‘523 variation on WMM in Black participants, and the first to examine this association using FW‐corrected metrics. These results support prior findings that ‘523‐S copy‐number is deleterious in White‐ε3/ε3 participants, but protective in Black‐ε4+ participants. These findings suggest a differential effect by racialized background of APOE on brain microstructural integrity in older adults.